# Supplementary material for: Michael Adduct of Sulfonamide Chalcone Targets Folate Metabolism in Brugia Malayi Parasite
Source: Biomedicines. 2023 Feb 27;11(3):723. doi: 10.3390/biomedicines11030723 (PMC10045823; doi:10.3390/biomedicines11030723)
Supplement: Supplementary file 1 [file biomedicines-11-00723-s001.zip › biomedicines-2168775-supplementary.pdf]

Supplementary Material

Spectra of Synthesized Michael Adducts

Figure S1: ^{1}H spectrum of 4-(4,4-dicyano-3-phenylbutanoyl)phenyl)benzenesulfonamide (3a) 2
Figure S2: ^{1}H spectrum of 4-(4,4-dicyano-3-phenylbutanoyl)phenyl)benzenesulfonamide (3a) 3
Figure S3: Mass spectrum of 4-(4,4-dicyano-3-phenylbutanoyl)phenyl)benzenesulfonamide (3a) 4
Figure S4: 1H spectrum of *N*-(4-(4,4-dicyano-3-(4-methoxyphenyl)butanoyl)phenyl)benzenesulfonamide (3b) 5
Figure S5: ^{13}C spectrum of *N*-(4-(4,4-dicyano-3-(4-methoxyphenyl)butanoyl)phenyl)benzenesulfonamide (3b) 6
Figure S6: Mass spectrum of *N*-(4-(4,4-dicyano-3-(4-methoxyphenyl)butanoyl)phenyl)benzenesulfonamide (3b) 7
Figure S7: ^{1}H spectrum of *N*-(4-(3-(4-chlorophenyl)-4,4-dicyanobutanoyl)phenyl)benzenesulfonamide (3c) 8
Figure S8: ^{13}C spectrum of *N*-(4-(3-(4-chlorophenyl)-4,4-dicyanobutanoyl)phenyl)benzenesulfonamide (3c) 9
Figure S9: Mass spectrum of *N*-(4-(3-(4-chlorophenyl)-4,4-dicyanobutanoyl)phenyl)benzenesulfonamide (3c) 10
Figure S10: ^{1}H spectrum of *N*-(4-(3-(4-bromophenyl)-4,4-dicyanobutanoyl)phenyl)benzenesulfonamide (3d) 11
Figure S11: ^{13}C spectrum of *N*-(4-(3-(4-bromophenyl)-4,4-dicyanobutanoyl)phenyl)benzenesulfonamide (3d) 12
Figure S12: Mass spectrum of *N*-(4-(3-(4-bromophenyl)-4,4-dicyanobutanoyl)phenyl)benzenesulfonamide (3d) 13
Figure S13: ^{1}H spectrum of *N*-(4-(4,4-dicyano-3-(3,4,5-trimethoxyphenyl)butanoyl)phenyl)benzene-sulfonamide (3e) 14
Figure S14: ^{13}C spectrum of *N*-(4-(4,4-dicyano-3-(3,4,5-trimethoxyphenyl)butanoyl)phenyl)benzene-sulfonamide (3e) 15
Figure S15: ^{1}H spectrum of *N*-(4-(3-(2-chlorophenyl)-4,4-dicyanobutanoyl)phenyl)benzenesulfonamide (3f) 16
Figure S16: ^{13}C spectrum of *N*-(4-(3-(2-chlorophenyl)-4,4-dicyanobutanoyl)phenyl)benzenesulfonamide (3f) 17
Figure S17: ^{1}H spectrum of *N*-(4-(4,4-dicyano-3-p-tolylbutanoyl)phenyl)benzenesulfonamide (3g) 18
Figure S18: ^{13}C spectrum of *N*-(4-(4,4-dicyano-3-p-tolylbutanoyl)phenyl)benzenesulfonamide (3g) 19
Figure S19: ^{1}H spectrum of *N*-(4-(4,4-dicyano-3-(4-methoxyphenyl)butanoyl)phenyl)-4 methylbenzene-sulfonamide 20
Figure S20: ^{13}C spectrum of *N*-(4-(4,4-dicyano-3-(4-methoxyphenyl)butanoyl)phenyl)-4 methylbenzene-sulfonamide 21
Figure S21: ^{1}H spectrum of *N*-(4-(3-(4-chlorophenyl)-4,4-dicyanobutanoyl)phenyl)-4-methylbenzenesulfonamide (3i) 22
Figure S22: ^{13}C spectrum of *N*-(4-(3-(4-chlorophenyl)-4,4-dicyanobutanoyl)phenyl)-4-methylbenzenesulfonamide (3i) 23
Figure S23: ^{1}H spectrum of *N*-(4-(3-(4-bromophenyl)-4,4-dicyanobutanoyl)phenyl)-4-methylbenzenesulfonamide (3j) 24
Figure S24: ^{13}C spectrum of *N*-(4-(3-(4-bromophenyl)-4,4-dicyanobutanoyl)phenyl)-4-methylbenzenesulfonamide (3j) 25
Figure S25: ^{1}H spectrum of *N*-(4-(4,4-dicyano-3-(4-isopropylphenyl)butanoyl)phenyl)-4-methylbenzenesulfonamide (3k) 26
Figure S26: ^{13}C spectrum of *N*-(4-(4,4-dicyano-3-(4-isopropylphenyl)butanoyl)phenyl)-4-methylbenzenesulfonamide (3k) 27
Figure S27: ^{1}H spectrum of *N*-(4-(3-(2-chlorophenyl)-4,4-dicyanobutanoyl)phenyl)-4-methylbenzenesulfonamide (3l) 28
Figure S28: ^{13}C spectrum of *N*-(4-(3-(2-chlorophenyl)-4,4-dicyanobutanoyl)phenyl)-4-methylbenzenesulfonamide (3l) 29
Figure S29: ^{1}H spectrum of *N*-(4-(4,4-dicyano-3-p-tolylbutanoyl)phenyl)-4-methylbenzenesulfonamide (3m) 30
Figure S30: ^{13}C spectrum of *N*-(4-(4,4-dicyano-3-p-tolylbutanoyl)phenyl)-4-methylbenzenesulfonamide (3m) 31
Figure S31: ^{1}H spectrum of *N*-(4-(3-(3-chlorophenyl)-4,4-dicyanobutanoyl)phenyl)-4-methylbenzenesulfonamide (3n) 32
Figure S32: ^{13}C spectrum of *N*-(4-(3-(3-chlorophenyl)-4,4-dicyanobutanoyl)phenyl)-4-methylbenzenesulfonamide (3n) 33

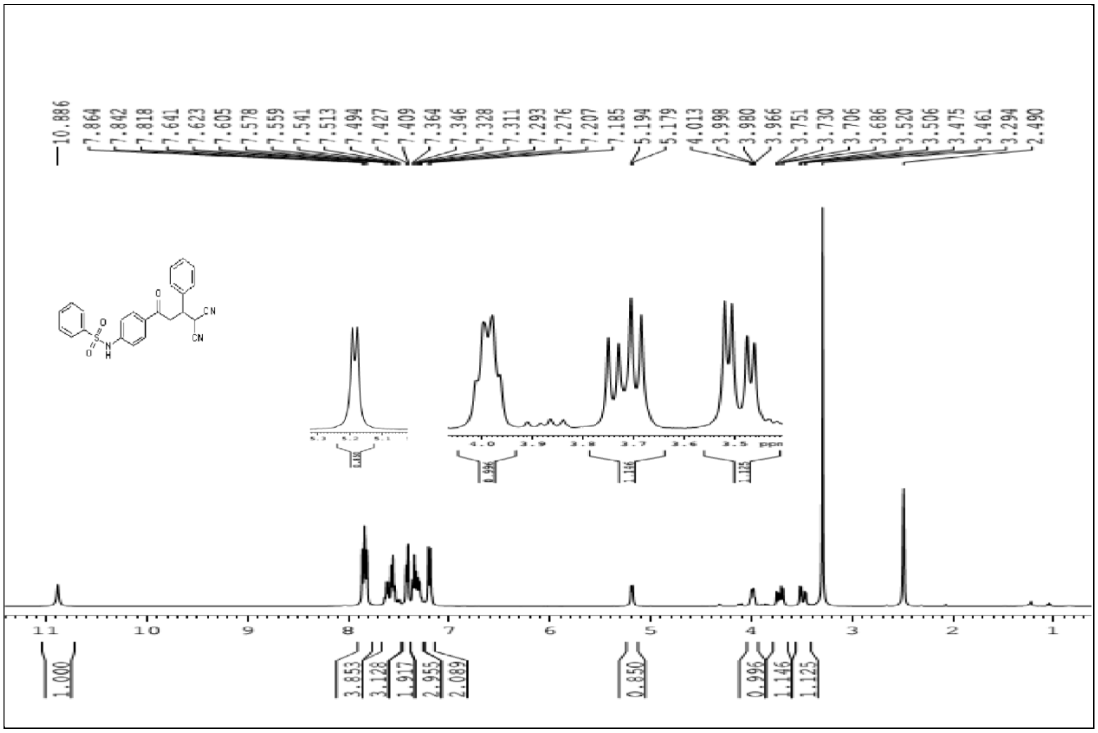

Figure S1. ^{1}H spectrum of 4-(4,4-dicyano-3-phenylbutanoyl)phenyl)benzenesulfonamide (3a).

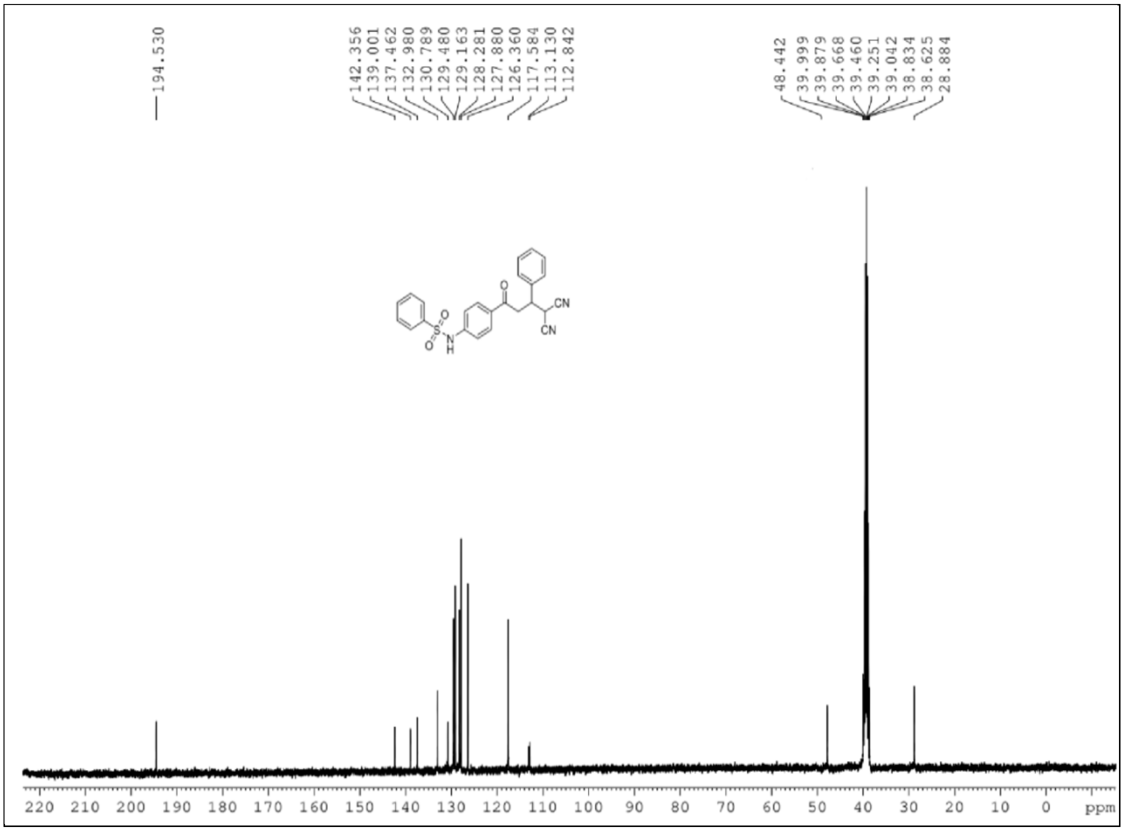

Figure S2. ^{1}H spectrum of 4-(4,4-dicyano-3-phenylbutanoyl)phenyl)benzenesulfonamide (3a).

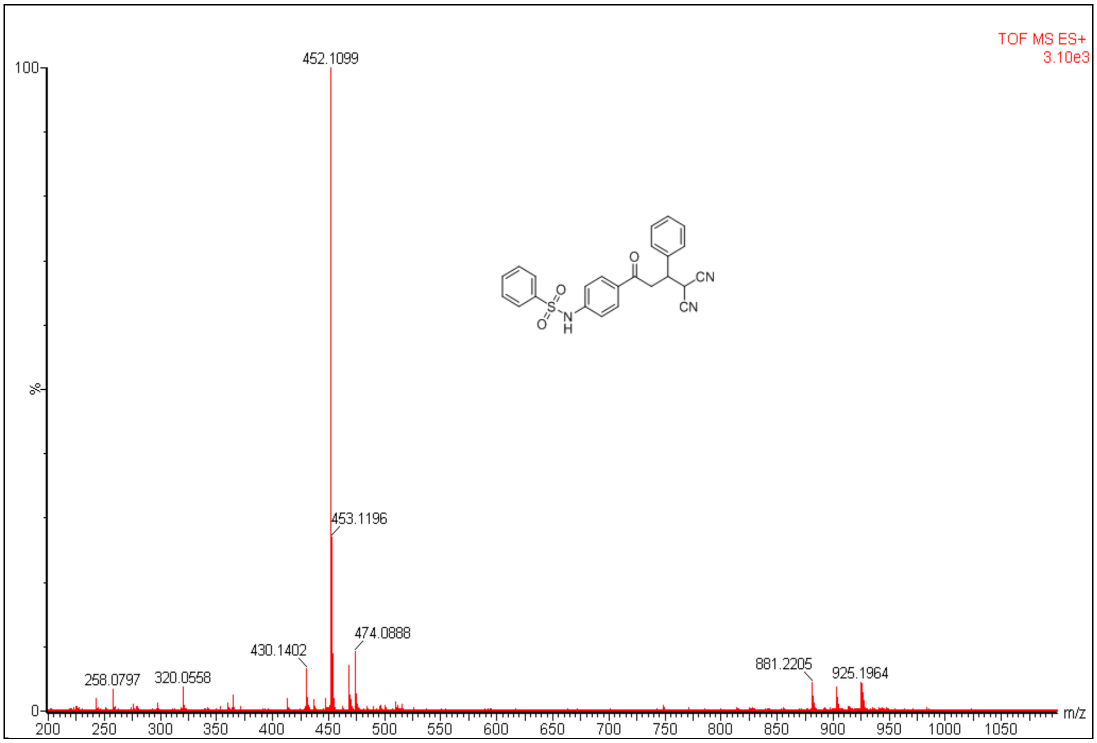

Figure S3. Mass spectrum of 4-(4,4-dicyano-3-phenylbutanoyl)phenyl)benzenesulfonamide (3a).

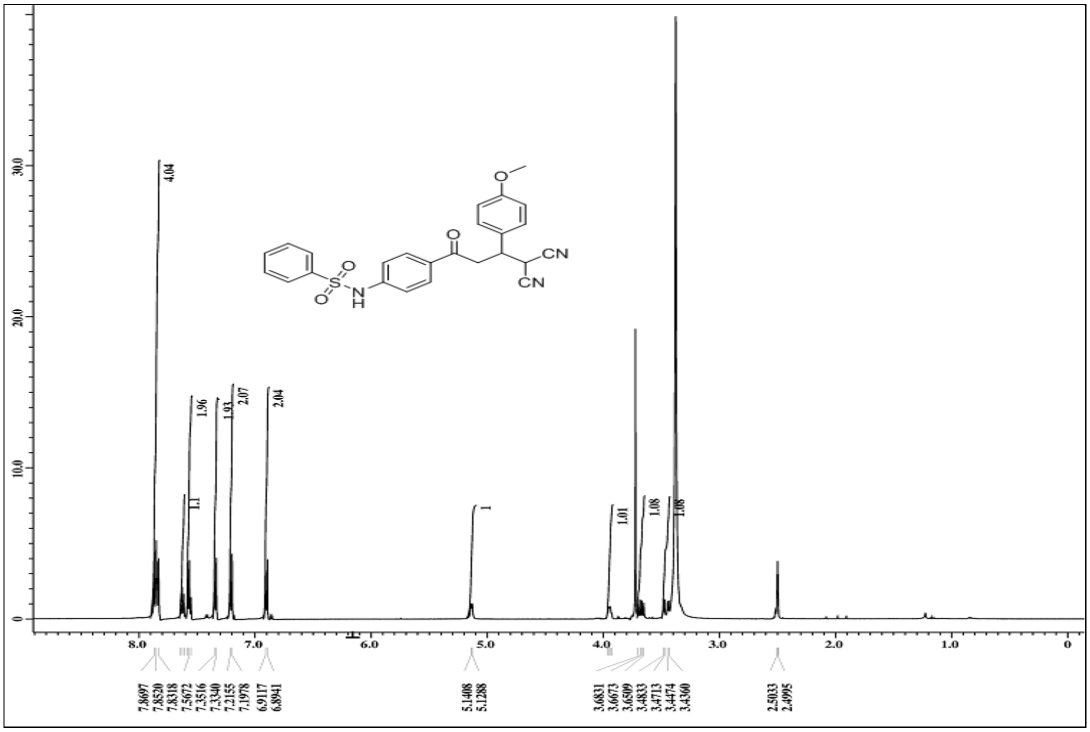

Figure S4. ^{1}H spectrum of N-(4-(4,4-dicyano-3-(4-methoxyphenyl)butanoyl)phenyl)benzenesulfonamide (3b).

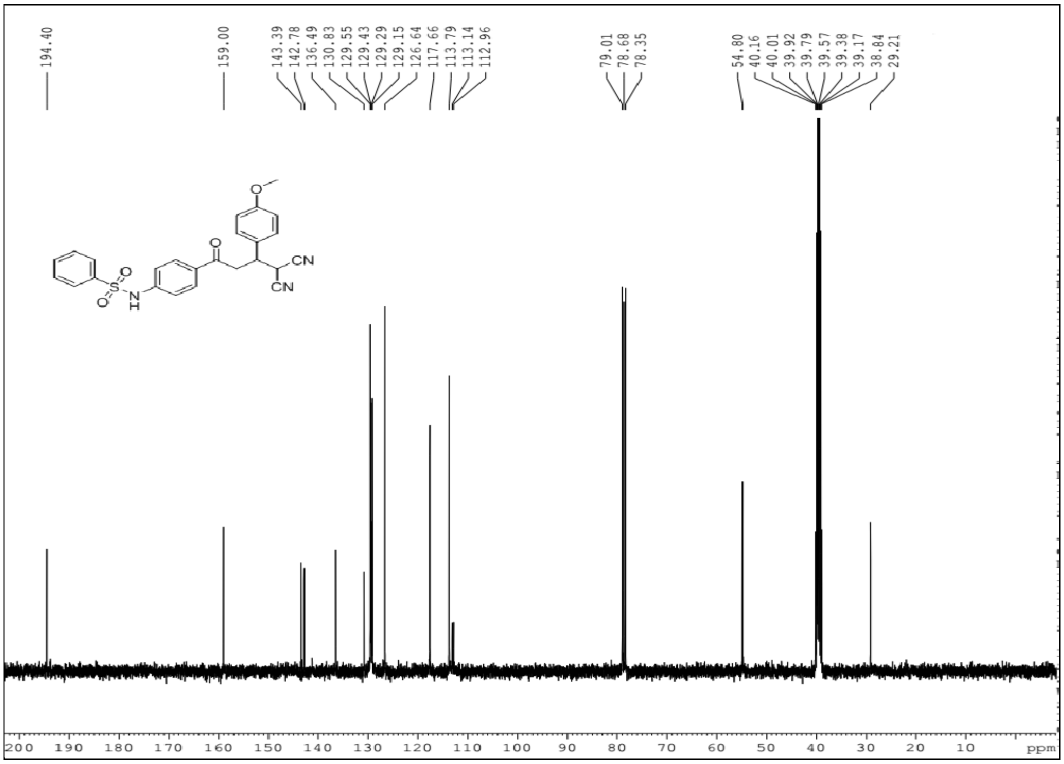

Figure S5. ^{13}C spectrum of N-(4-(4,4-dicyano-3-(4-methoxyphenyl)butanoyl)phenyl)benzenesulfonamide (3b).

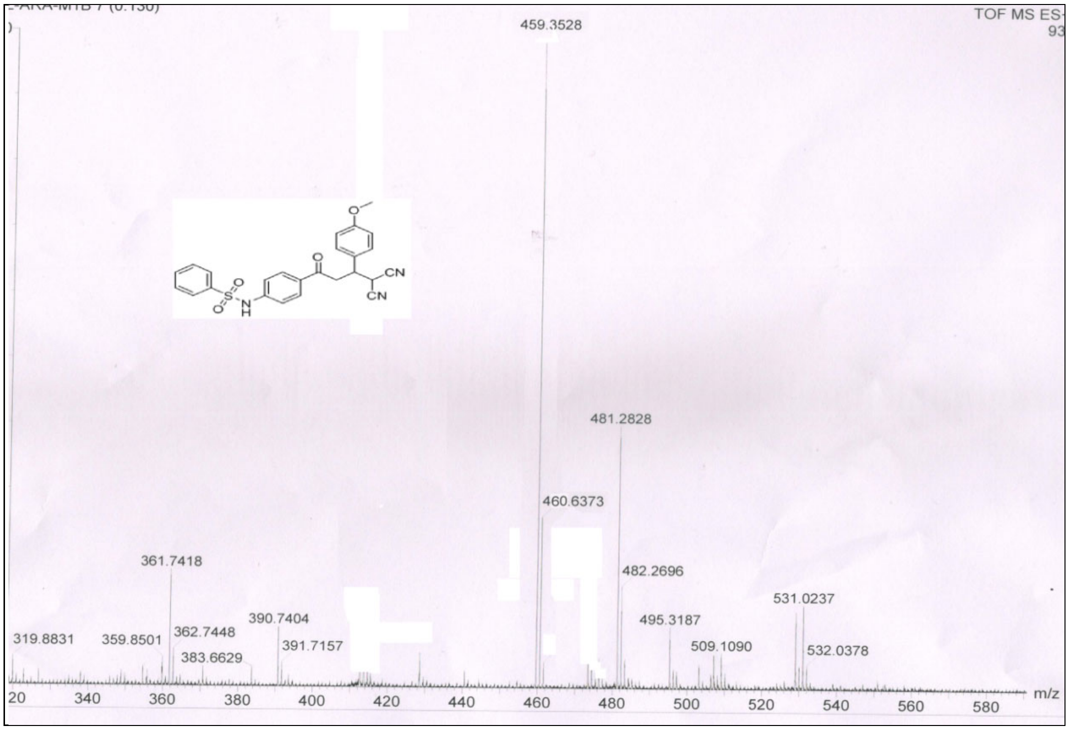

Figure S6. Mass spectrum of N-(4-(4,4-dicyano-3-(4-methoxyphenyl)butanoyl)phenyl)benzenesulfonamide (3b).

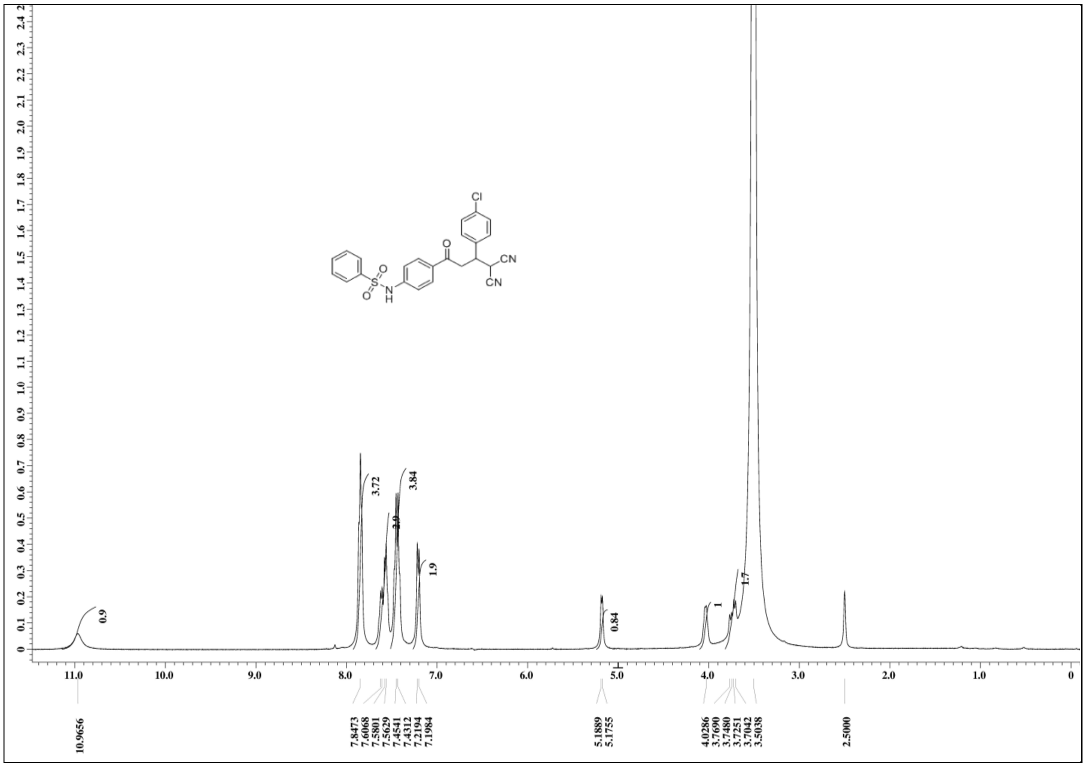

Figure S7. ^{1}H spectrum of N-(4-(3-(4-chlorophenyl)-4,4-dicyanobutanoyl)phenyl)benzenesulfonamide (3c).

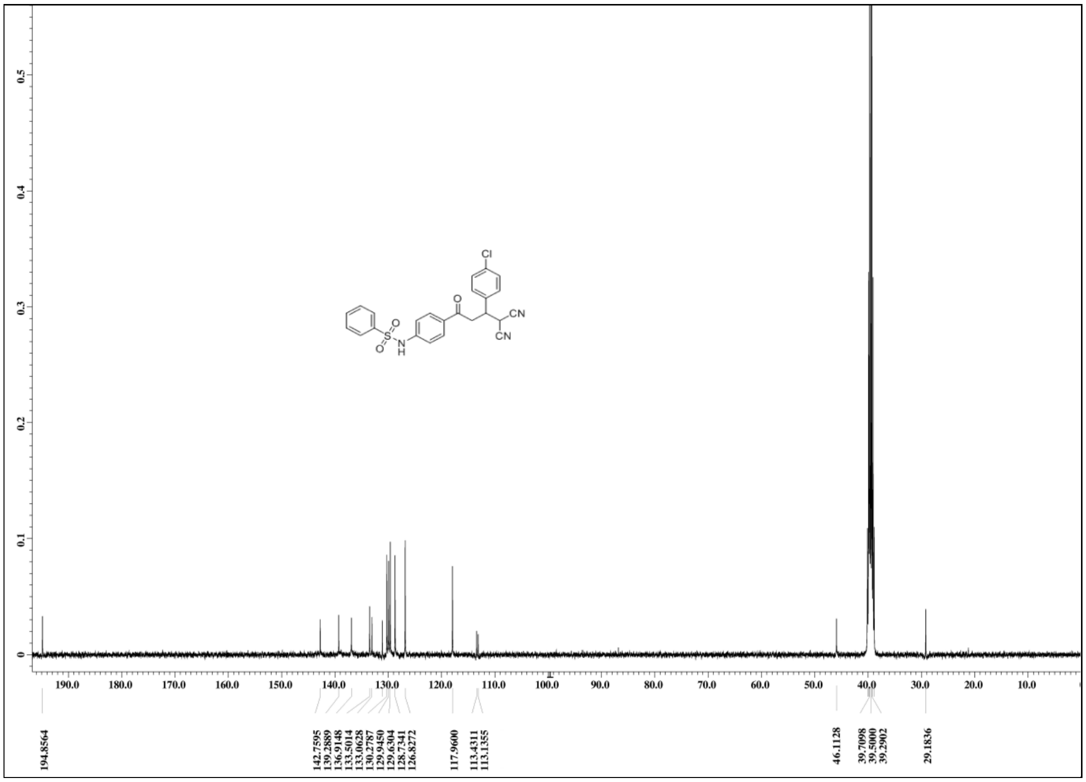

Figure S8. ^{13}C spectrum of N-(4-(3-(4-chlorophenyl)-4,4-dicyanobutanoyl)phenyl)benzenesulfonamide (3c).

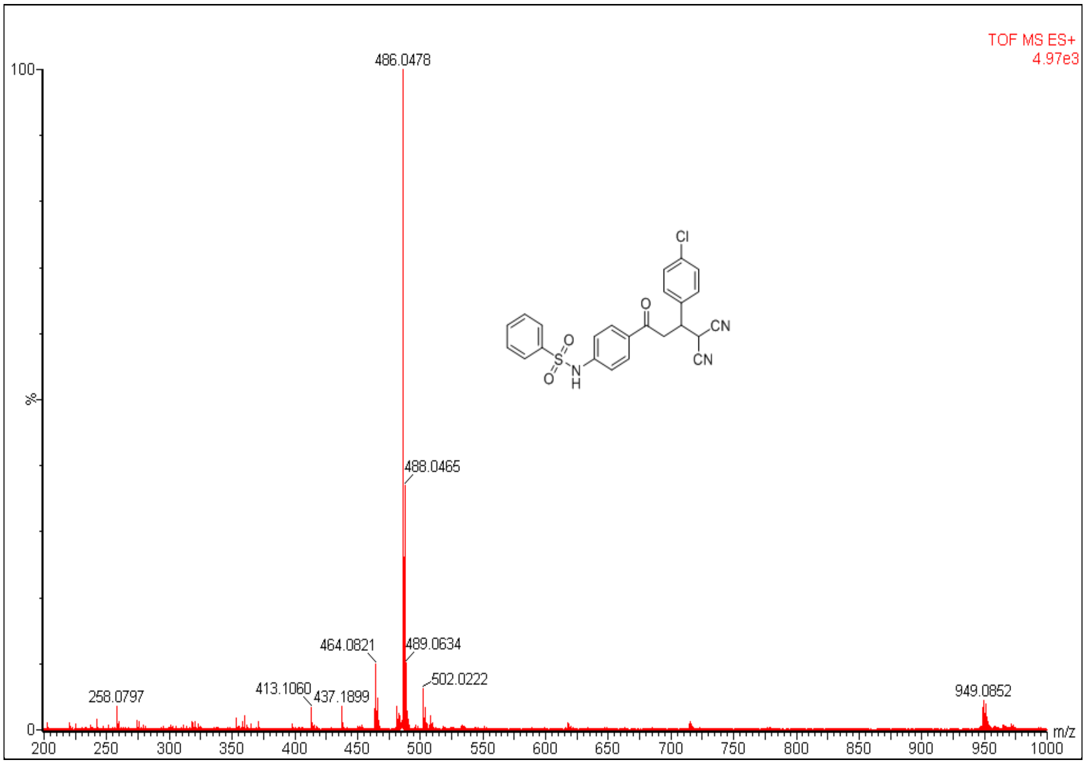

Figure S9. Mass spectrum of N-(4-(3-(4-chlorophenyl)-4,4-dicyanobutanoyl)phenyl)benzenesulfonamide (3c).

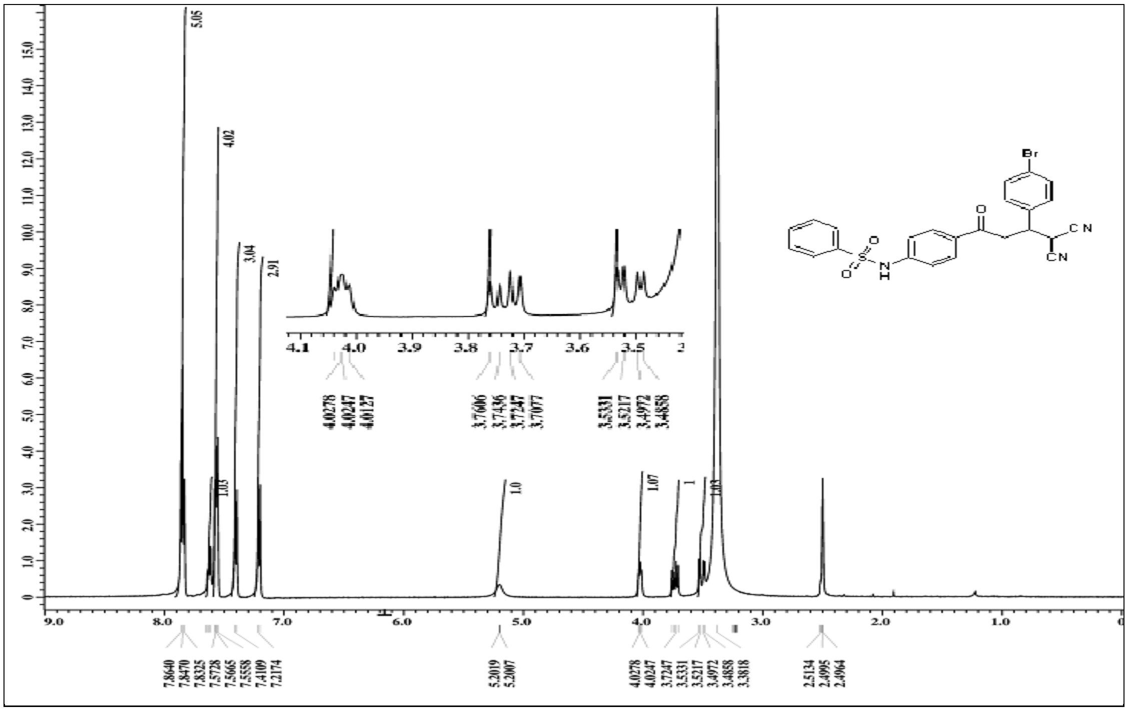

Figure S10. ^{1}H spectrum of N-(4-(3-(4-bromophenyl)-4,4-dicyanobutanoyl)phenyl)benzenesulfonamide (3d).

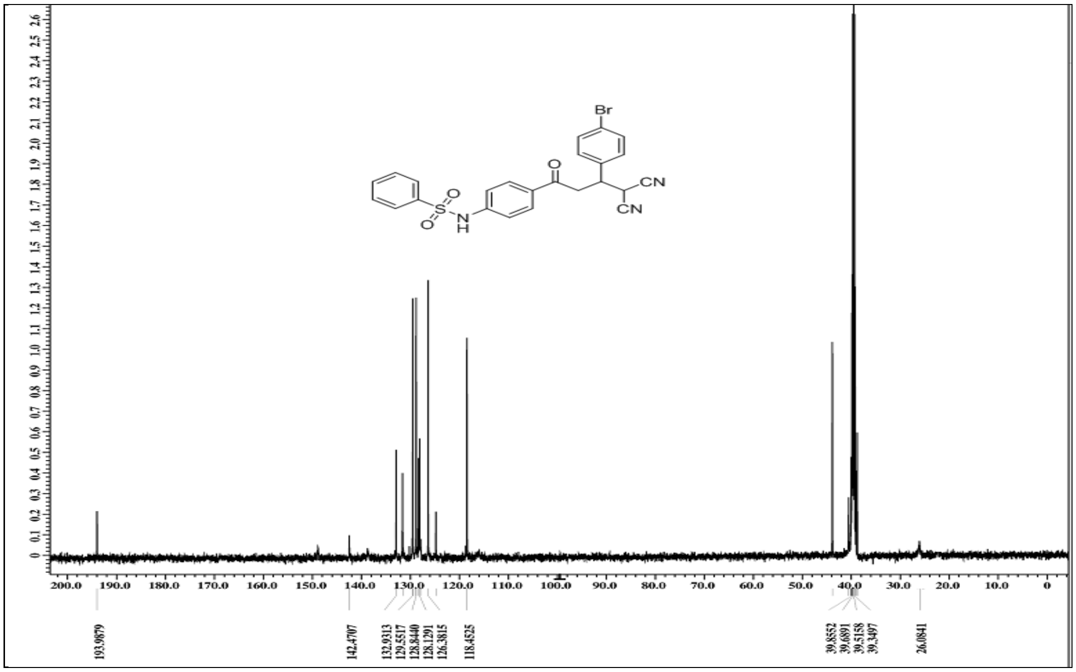

Figure S11. ^{13}C spectrum of N-(4-(3-(4-bromophenyl)-4,4-dicyanobutanoyl)phenyl)benzenesulfonamide (3d).

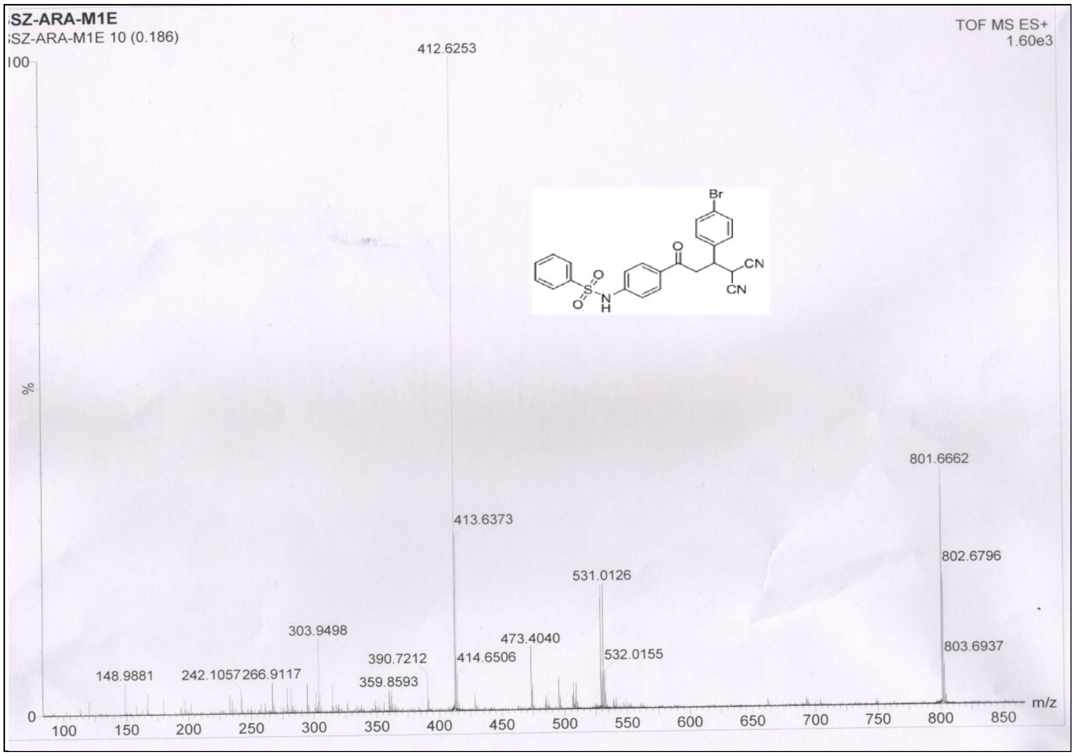

Figure S12. Mass spectrum of N-(4-(3-(4-bromophenyl)-4,4-dicyanobutanoyl)phenyl)benzenesulfonamide (3d).

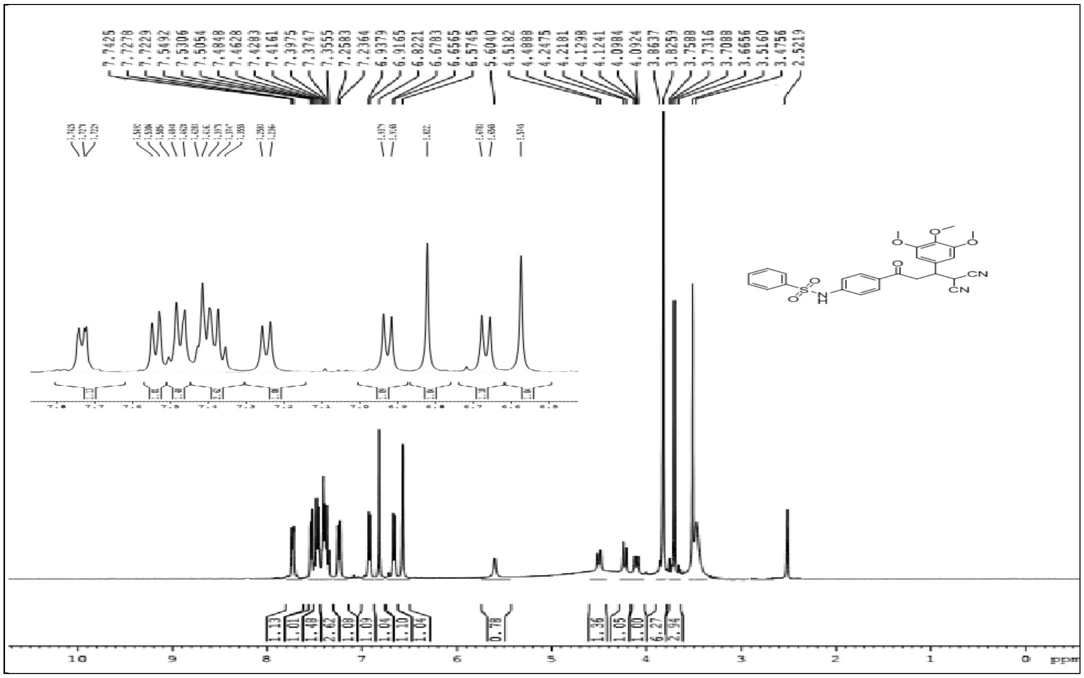

Figure S13. ^{1}H spectrum of N-(4-(4,4-dicyano-3-(3,4,5-trimethoxyphenyl)butanoyl)phenyl)benzene-sulfonamide (3e).

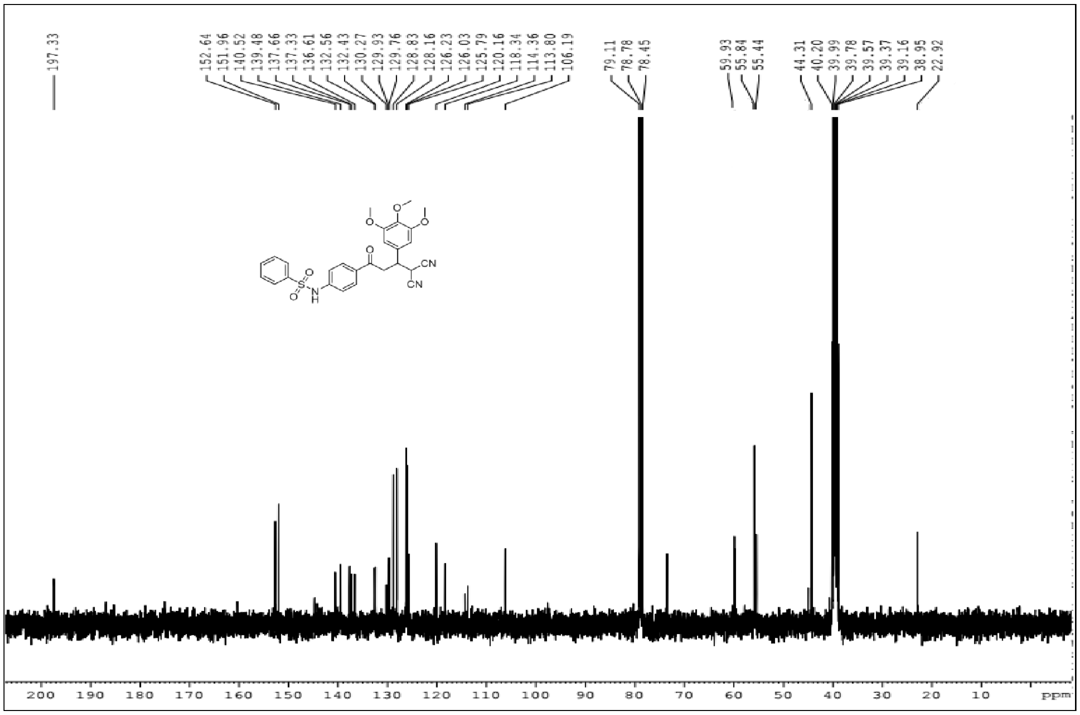

Figure S14. ^{13}C spectrum of N-(4-(4,4-dicyano-3-(3,4,5-trimethoxyphenyl)butanoyl)phenyl)benzene-sulfonamide (3e).

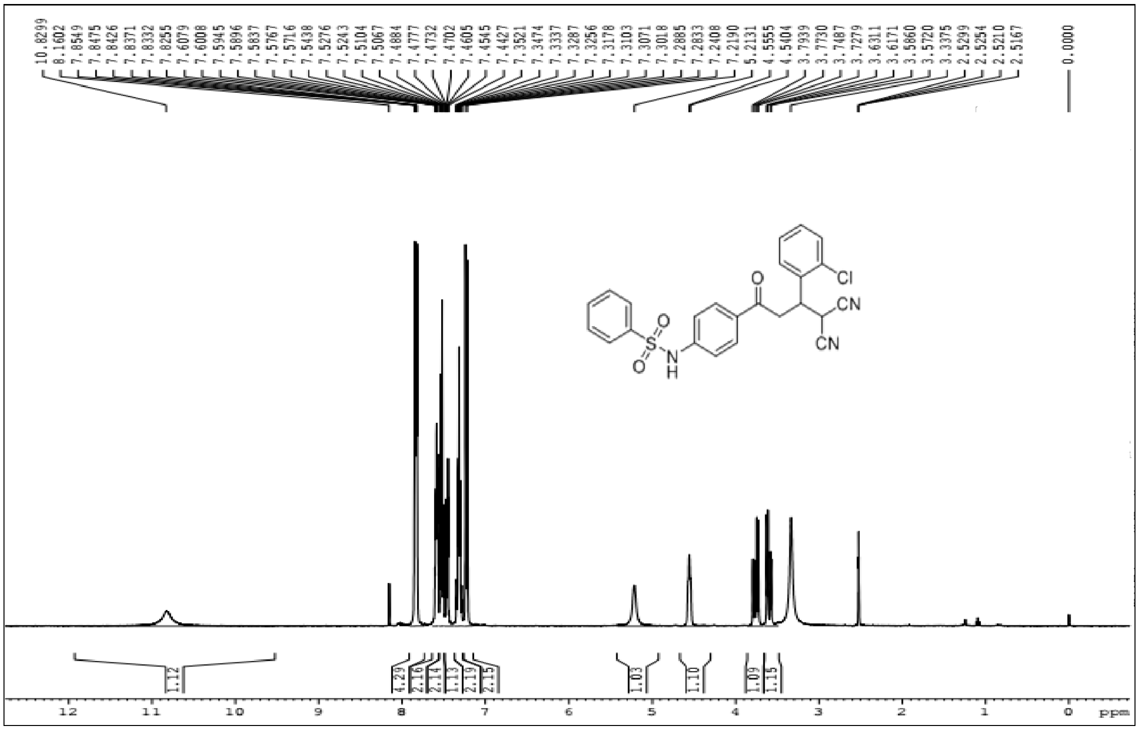

Figure S15. ^{1}H spectrum of N-(4-(3-(2-chlorophenyl)-4,4-dicyanobutanoyl)phenyl)benzenesulfonamide (3f).

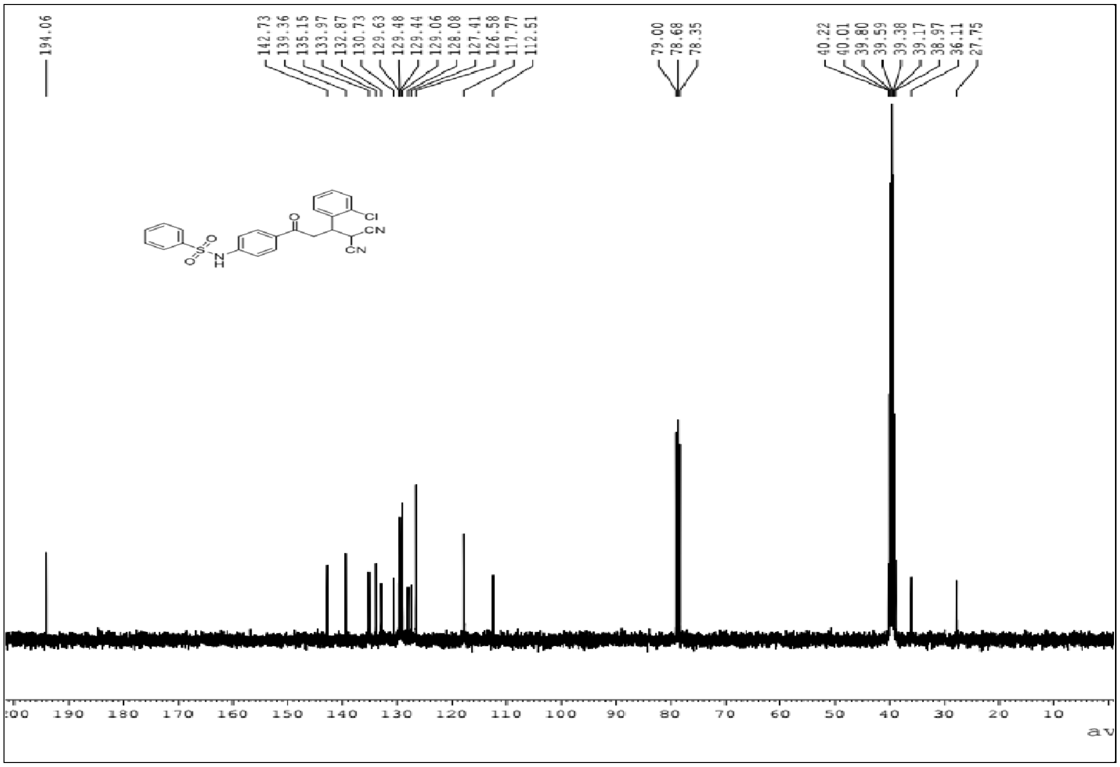

Figure S16. ^{13}C spectrum of N-(4-(3-(2-chlorophenyl)-4,4-dicyanobutanoyl)phenyl)benzenesulfonamide (3f).

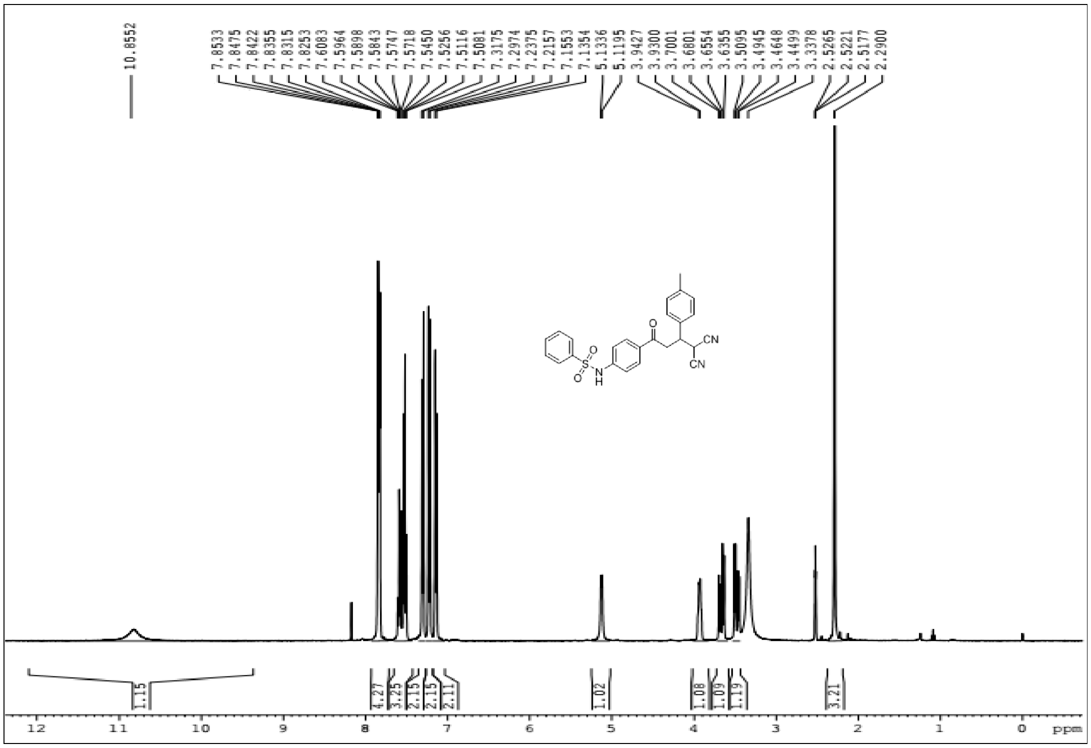

Figure S17. ^{1}H spectrum of N-(4-(4,4-dicyano-3-p-tolylbutanoyl)phenyl)benzenesulfonamide (3g).

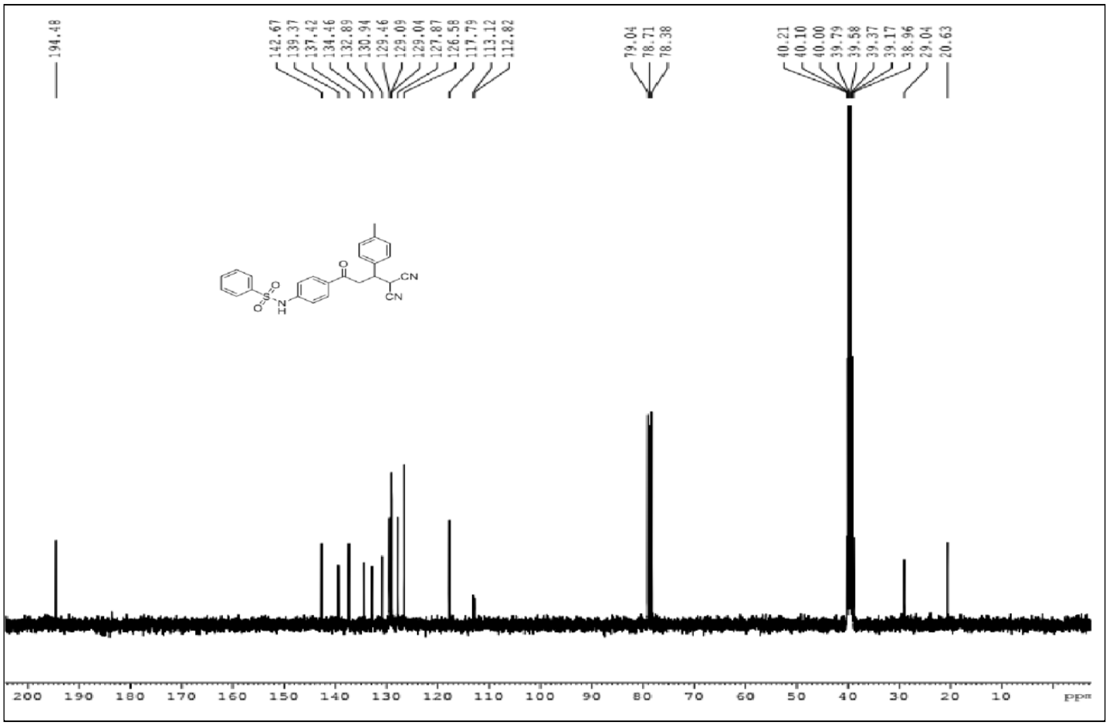

Figure S18. ^{13}C spectrum of N-(4-(4,4-dicyano-3-p-tolylbutanoyl)phenyl)benzenesulfonamide (3g).

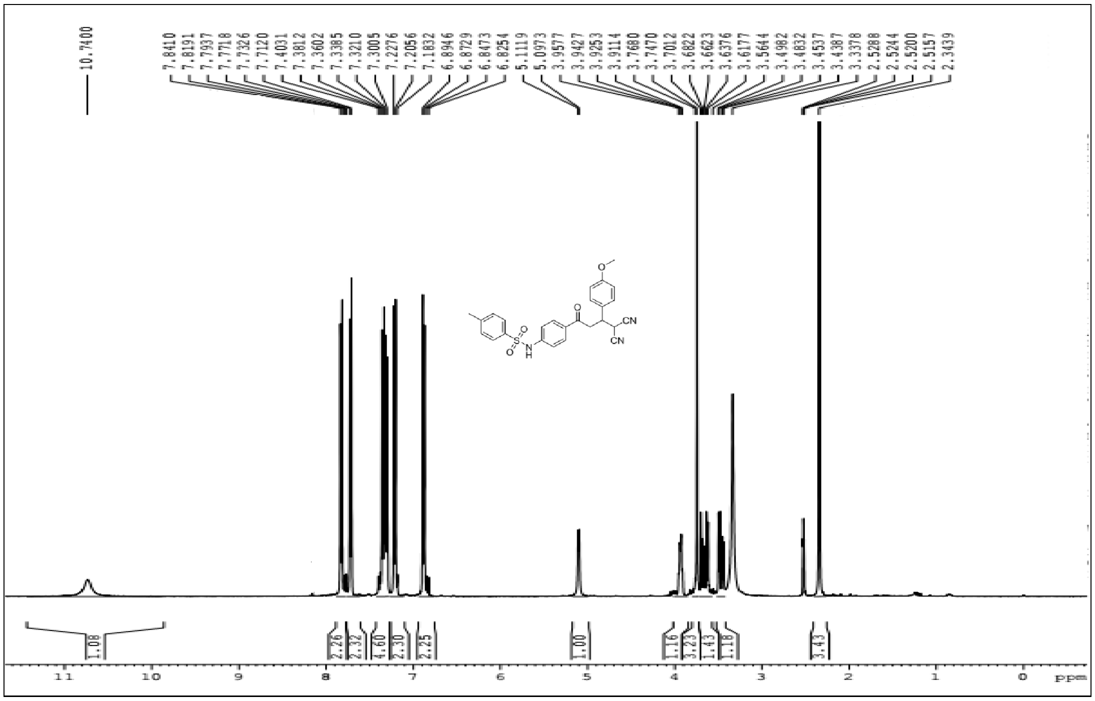

Figure S19. ^{1}H spectrum of N-(4-(4,4-dicyano-3-(4-methoxyphenyl)butanoyl)phenyl)-4 methylbenzene-sulfonamide (3h).

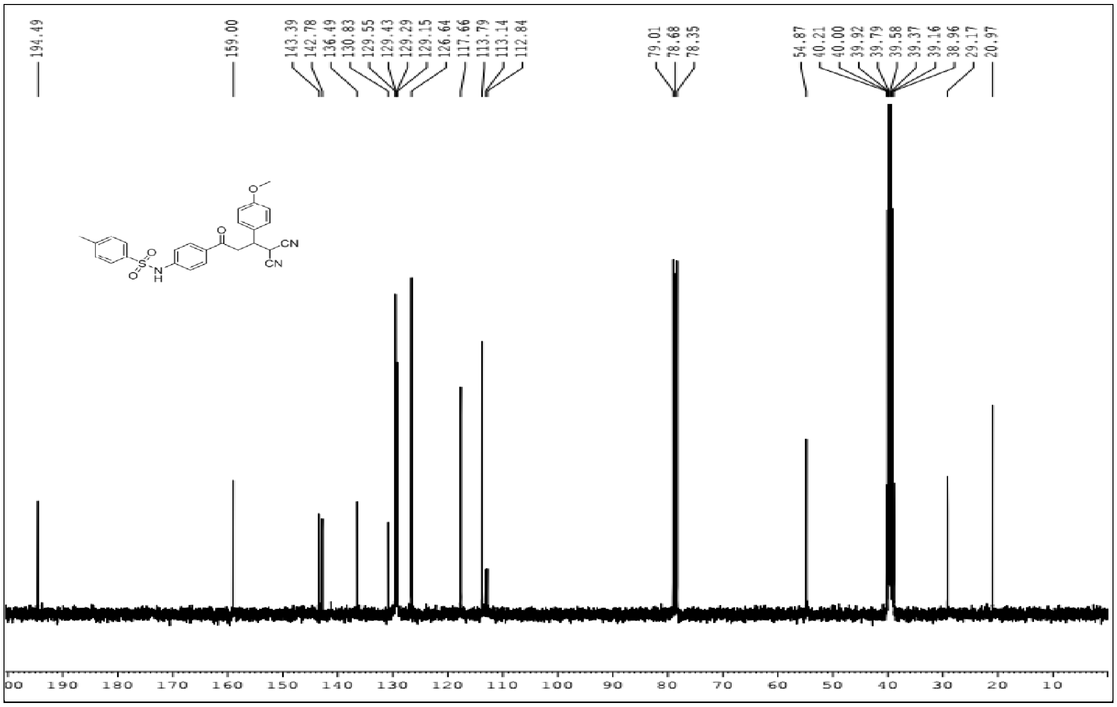

Figure S20. ^{13}C spectrum of N-(4-(4,4-dicyano-3-(4-methoxyphenyl)butanoyl)phenyl)-4 methylbenzene-sulfonamide (3h).

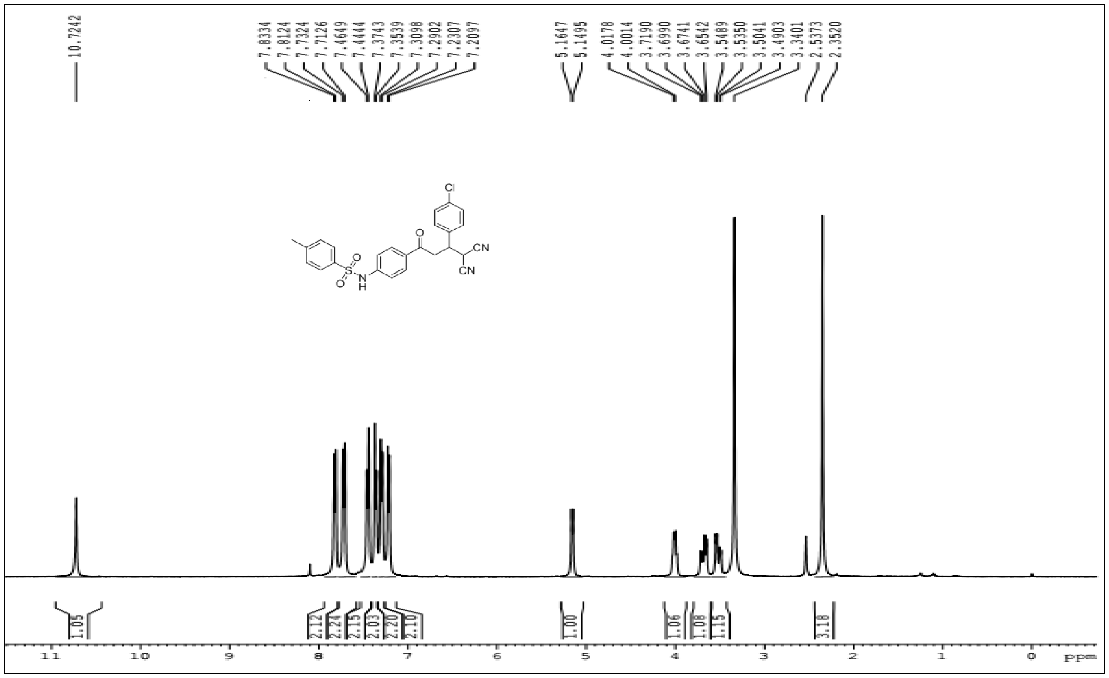

Figure S21. ^{1}H spectrum of N-(4-(3-(4-chlorophenyl)-4,4-dicyanobutanoyl)phenyl)-4-methylbenzenesulfonamide (3i).

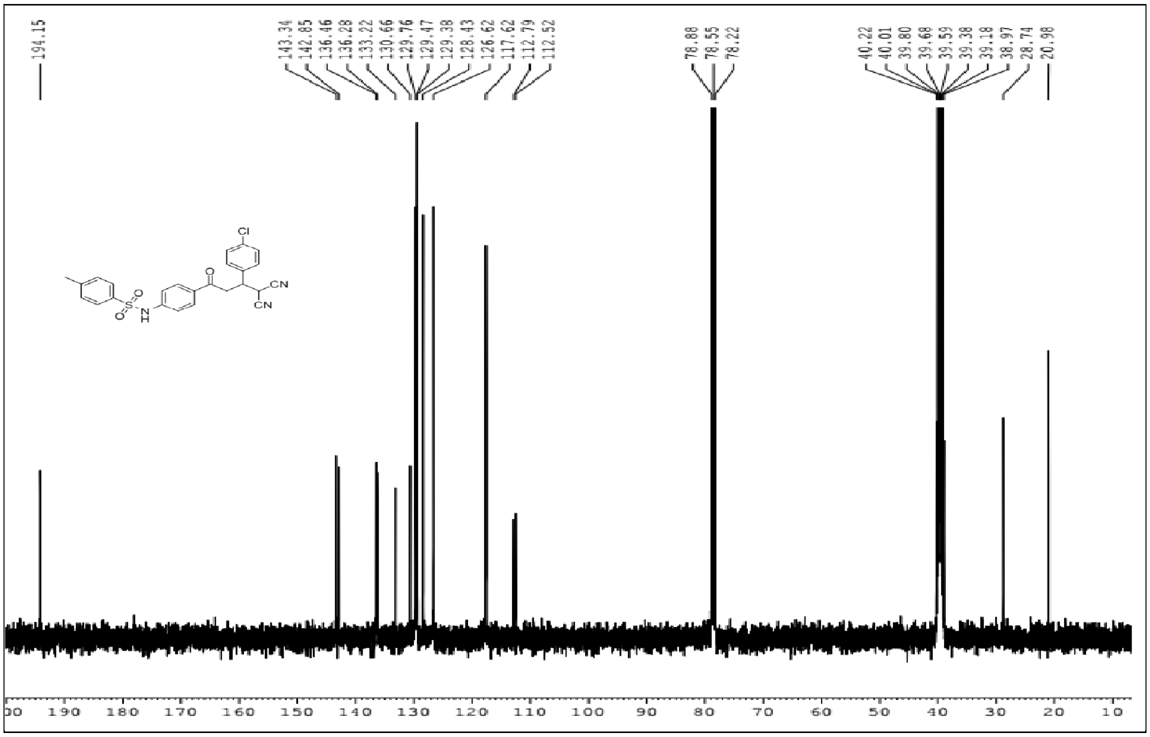

Figure S22. ^{13}C spectrum of N-(4-(3-(4-chlorophenyl)-4,4-dicyanobutanoyl)phenyl)-4-methylbenzenesulfonamide (3i).

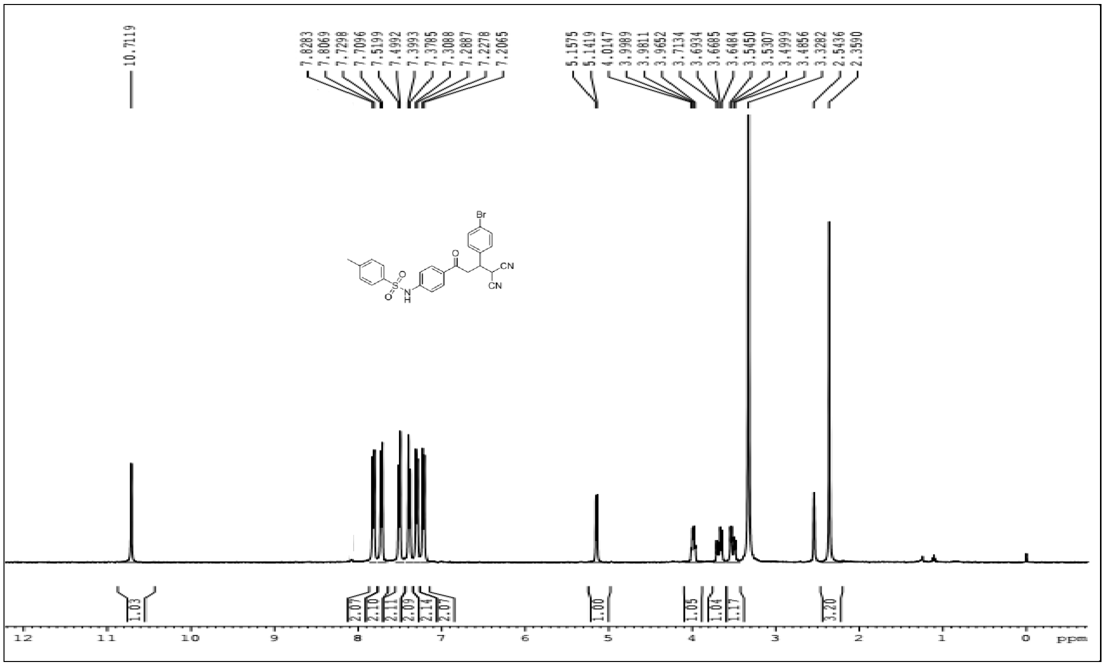

Figure S23. ^{1}H spectrum of N-(4-(3-(4-bromophenyl)-4,4-dicyanobutanoyl)phenyl)-4-methylbenzenesulfonamide (3j).

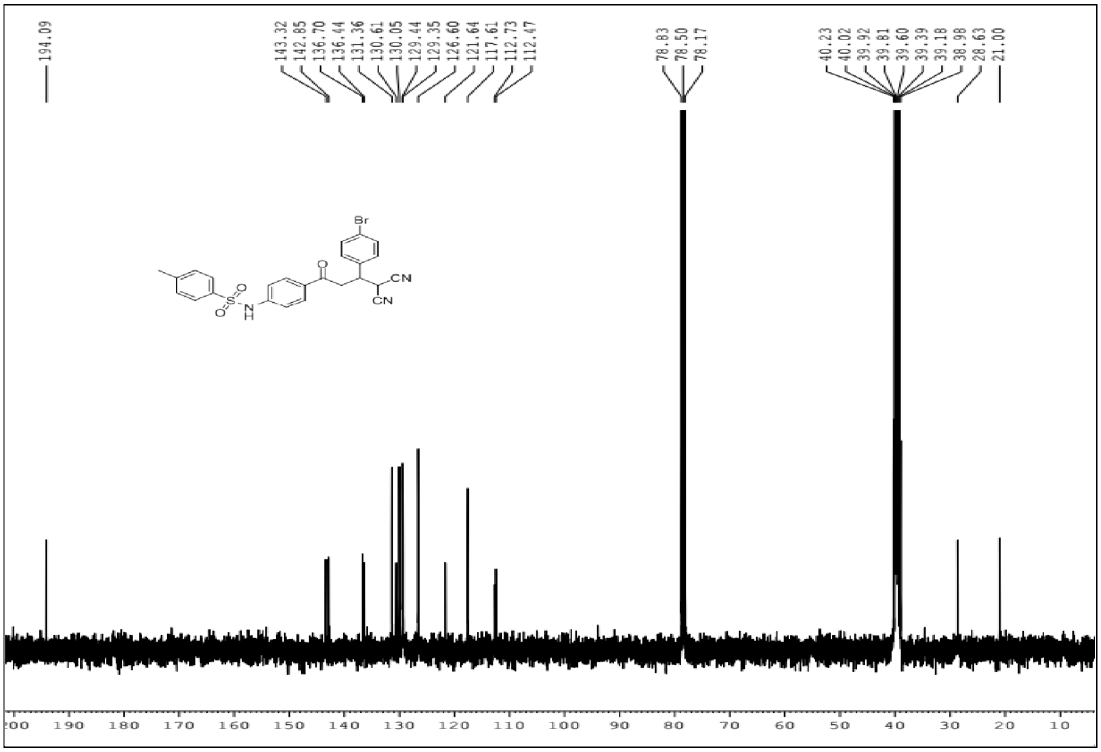

Figure S24. ^{13}C spectrum of N-(4-(3-(4-bromophenyl)-4,4-dicyanobutanoyl)phenyl)-4-methylbenzenesulfonamide (3j).

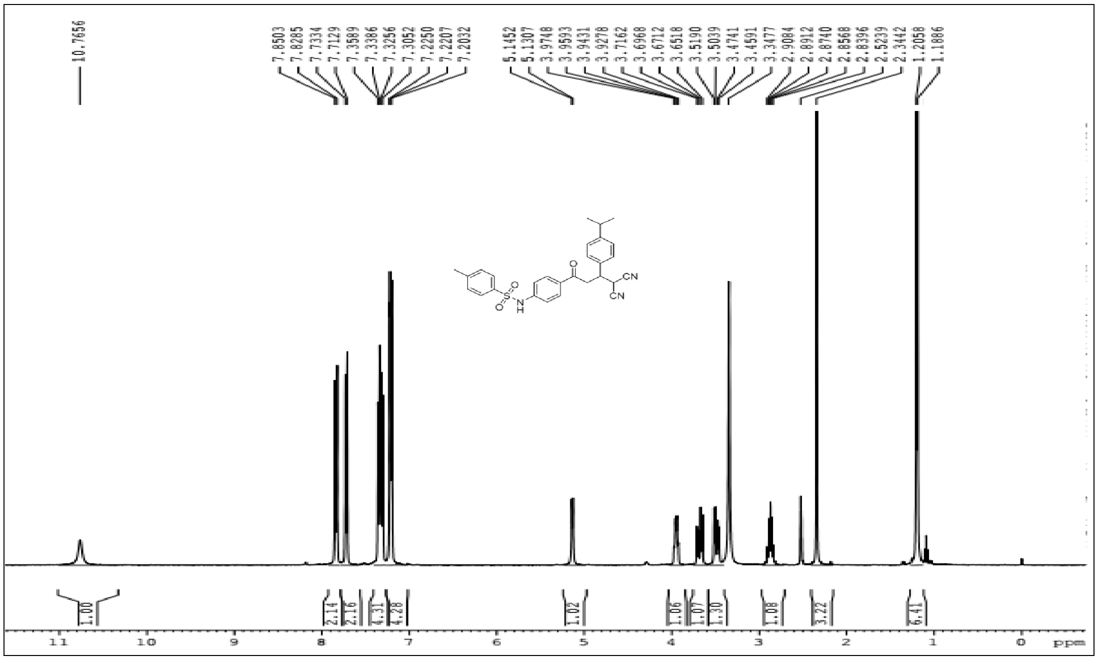

Figure S25. ^{1}H spectrum of N-(4-(4,4-dicyano-3-(4-isopropylphenyl)butanoyl)phenyl)-4-methylbenzenesulfonamide (3k).

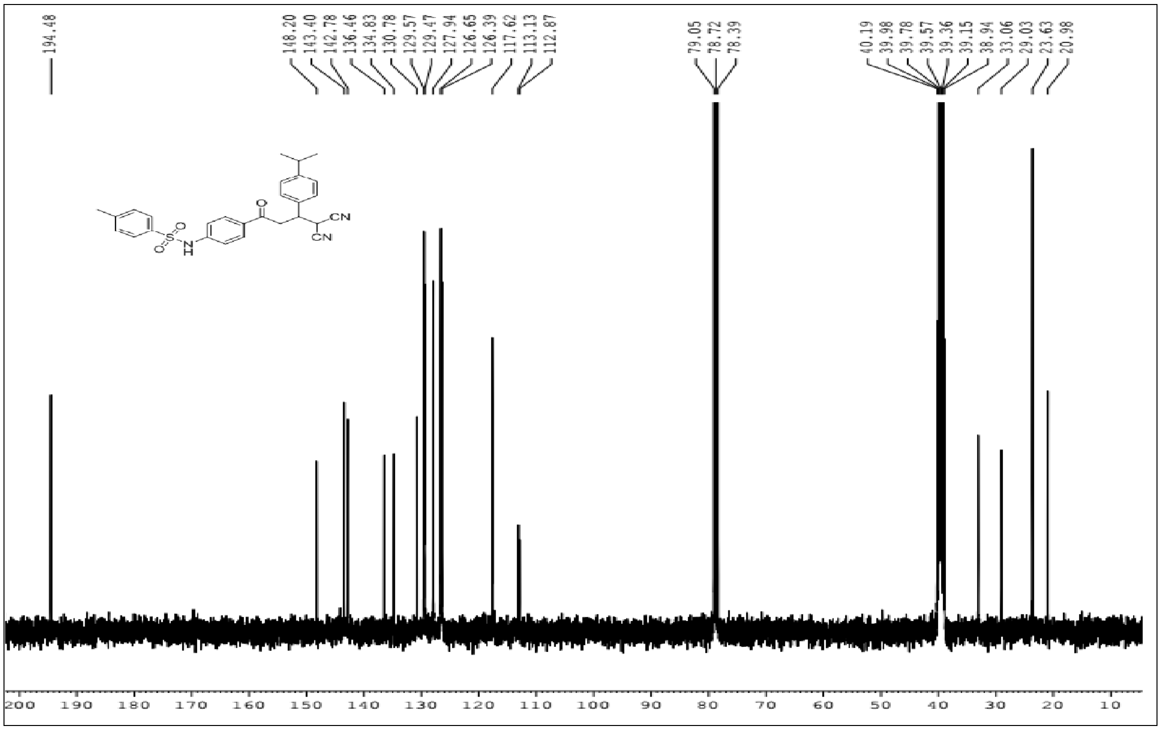

Figure S26. ^{13}C spectrum of *N*-(4-(4,4-dicyano-3-(4-isopropylphenyl)butanoyl)phenyl)-4-methylbenzenesulfonamide (3k).

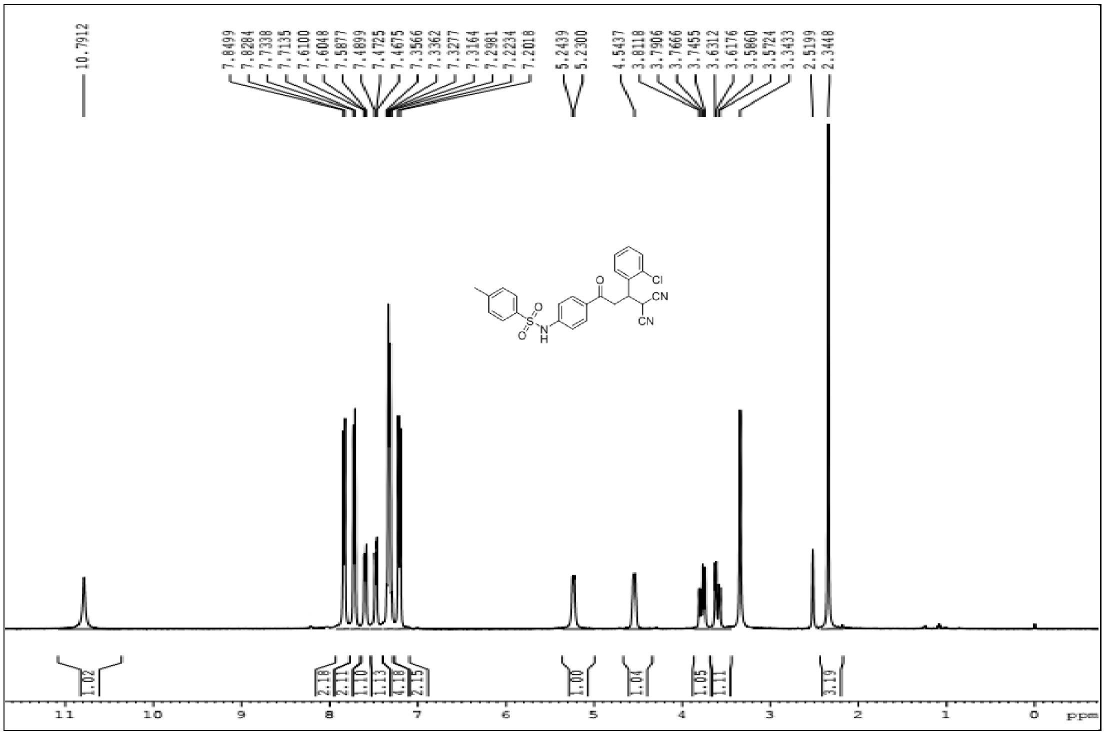

Figure S27. ^{1}H spectrum of N-(4-(3-(2-chlorophenyl)-4,4-dicyanobutanoyl)phenyl)-4-methylbenzenesulfonamide (3l).

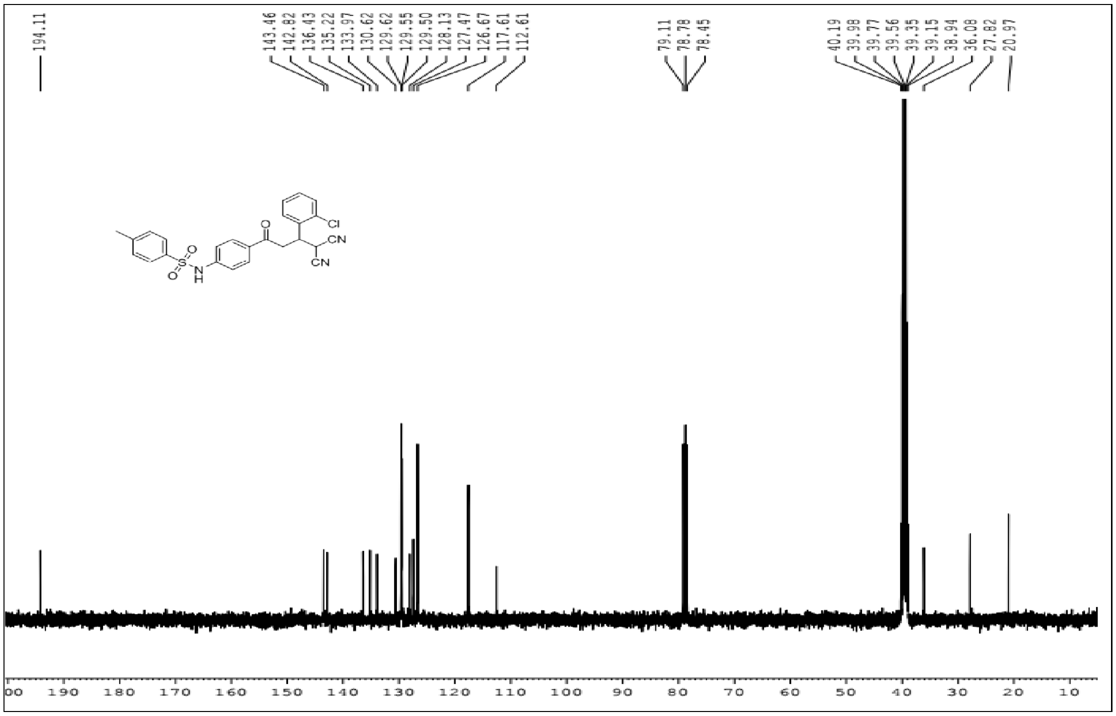

Figure S28. ^{13}C spectrum of N-(4-(3-(2-chlorophenyl)-4,4-dicyanobutanoyl)phenyl)-4-methylbenzenesulfonamide (3l).

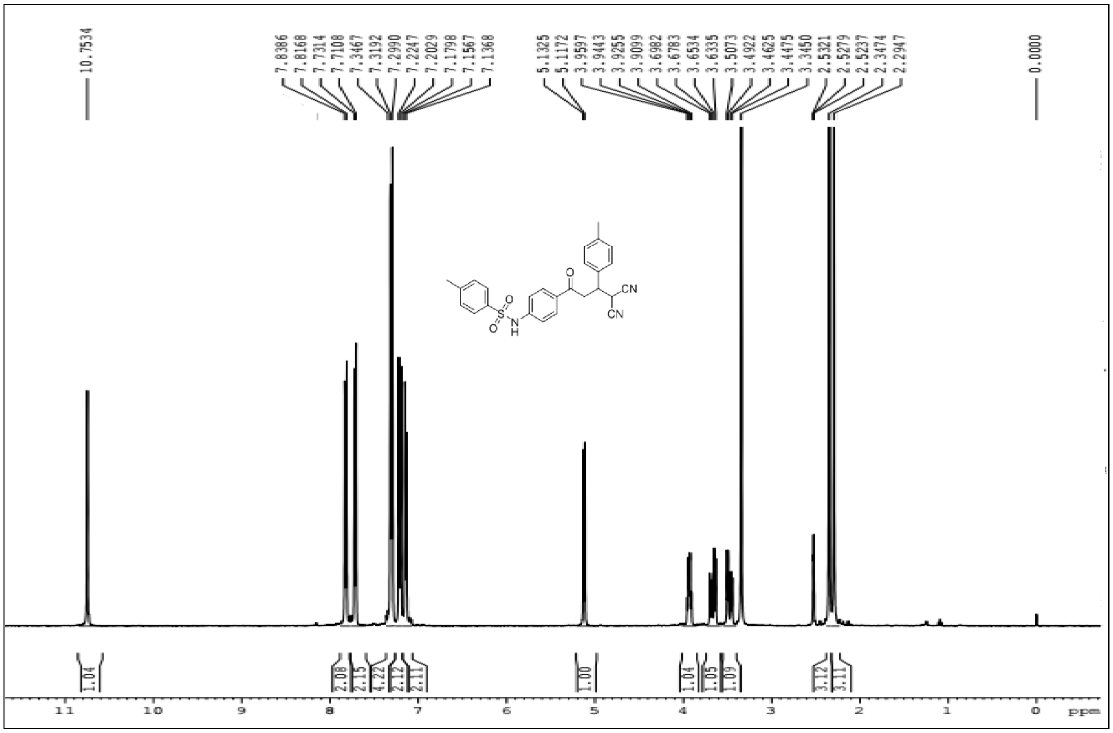

Figure S29. ^{1}H spectrum of N-(4-(4,4-dicyano-3-p-tolylbutanoyl)phenyl)-4-methylbenzenesulfonamide (3m).

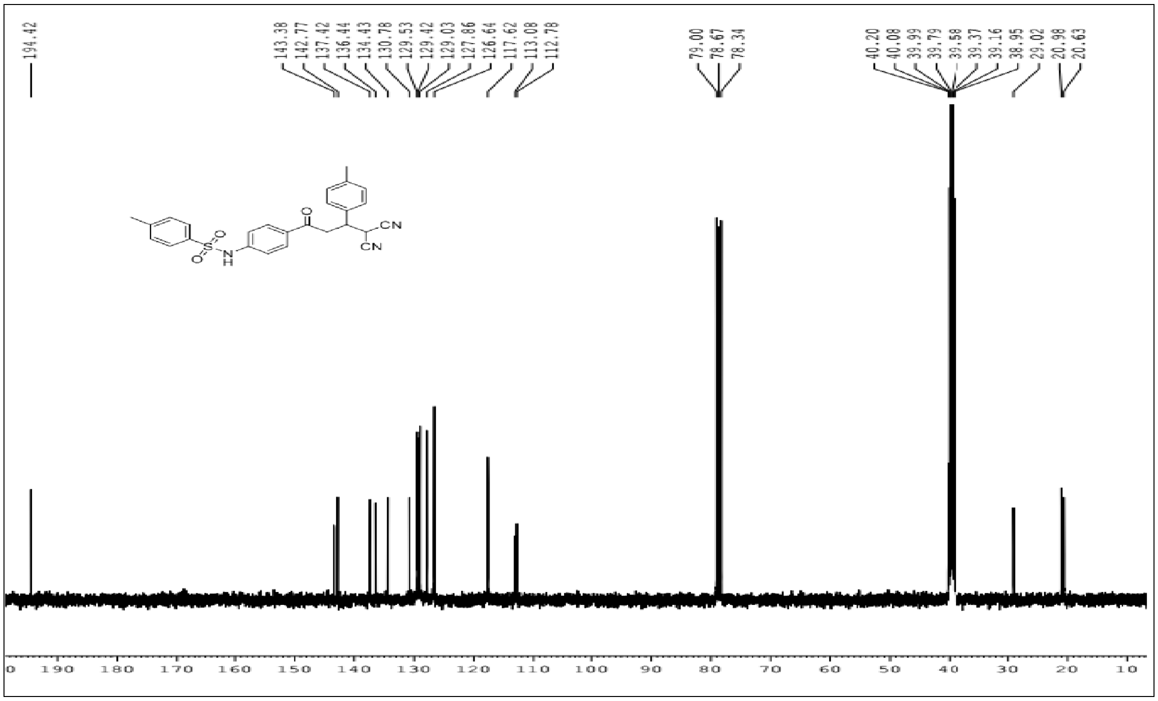

Figure S30. ^{13}C spectrum of N-(4-(4,4-dicyano-3-p-tolylbutanoyl)phenyl)-4-methylbenzenesulfonamide (3m).

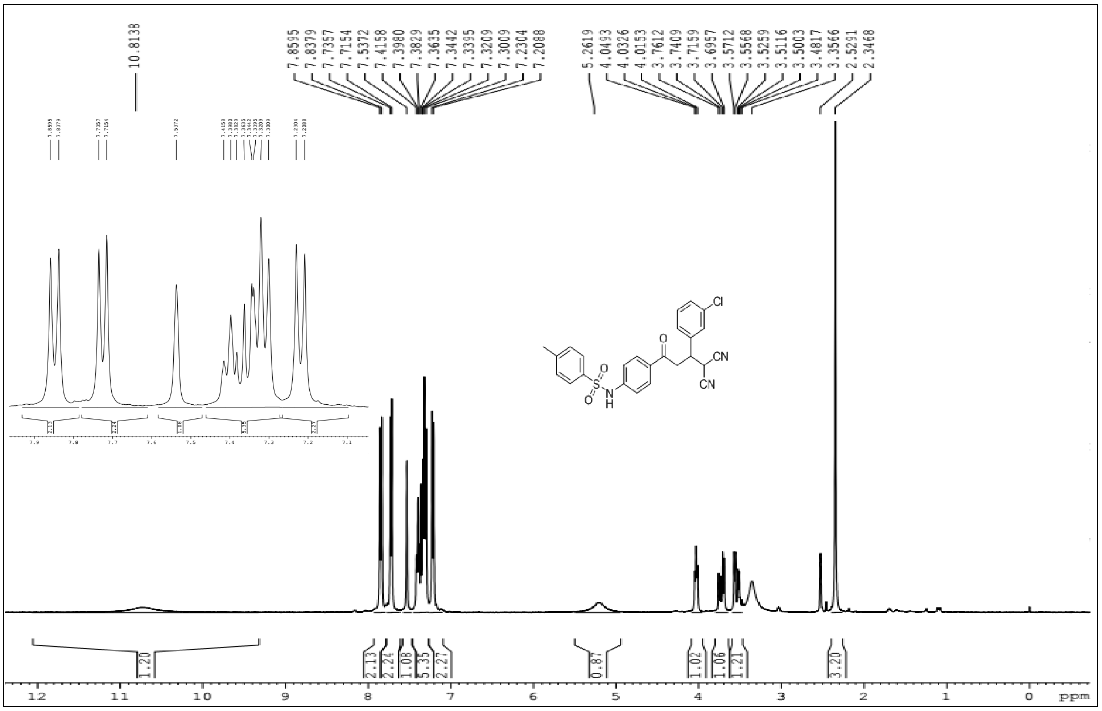

Figure S31. ^{1}H spectrum of N-(4-(3-(3-chlorophenyl)-4,4-dicyanobutanoyl)phenyl)-4-methylbenzenesulfonamide (3n).

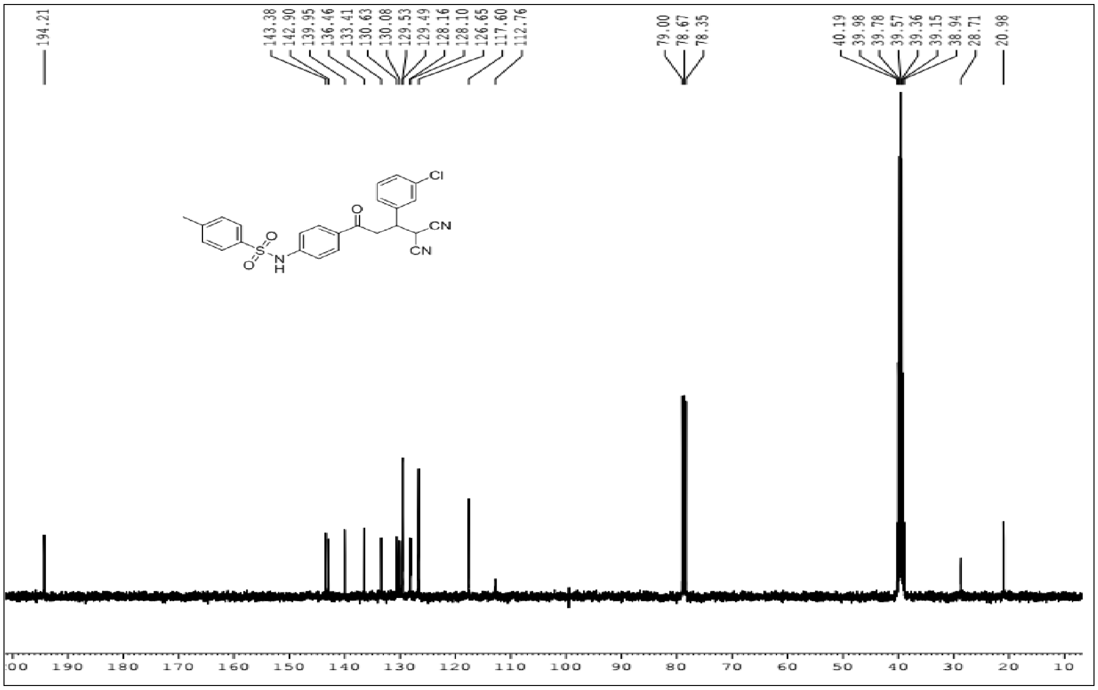

Figure S32. ^{13}C spectrum of N-(4-(3-(3-chlorophenyl)-4,4-dicyanobutanoyl)phenyl)-4-methylbenzenesulfonamide (3n).
